# Analysis of polygenic selection in purebred and crossbred pig genomes using generation proxy selection mapping

**DOI:** 10.1186/s12711-023-00836-9

**Published:** 2023-09-14

**Authors:** Caleb J. Grohmann, Caleb M. Shull, Tamar E. Crum, Clint Schwab, Timothy J. Safranski, Jared E. Decker

**Affiliations:** 1 University of Missouri, Columbia, MO 65211 USA; 2 The Maschhoff’s, LLC, Carlyle, IL 62231 USA

## Abstract

**Background:**

Artificial selection on quantitative traits using breeding values and selection indices in commercial livestock breeding populations causes changes in allele frequency over time at hundreds or thousands of causal loci and the surrounding genomic regions. In population genetics, this type of selection is called polygenic selection. Researchers and managers of pig breeding programs are motivated to understand the genetic basis of phenotypic diversity across genetic lines, breeds, and populations using selection mapping analyses. Here, we applied generation proxy selection mapping (GPSM), a genome-wide association analysis of single nucleotide polymorphism (SNP) genotypes (38,294–46,458 markers) of birth date, in four pig populations (15,457, 15,772, 16,595 and 8447 pigs per population) to identify loci responding to artificial selection over a period of five to ten years. Gene-drop simulation analyses were conducted to provide context for the GPSM results. Selected loci within and across each population of pigs were compared in the context of swine breeding objectives.

**Results:**

The GPSM identified 49 to 854 loci as under selection (*Q*-values less than 0.10) across 15 subsets of pigs based on combinations of populations. The number of significant associations increased when data were pooled across populations. In addition, several significant associations were identified in more than one population. These results indicate concurrent selection objectives, similar genetic architectures, and shared causal variants responding to selection across these pig populations. Negligible error rates (less than or equal to 0.02%) of false-positive associations were found when testing GPSM on gene-drop simulated genotypes, suggesting that GPSM distinguishes selection from random genetic drift in actual pig populations.

**Conclusions:**

This work confirms the efficacy and the negligible error rates of the GPSM method in detecting selected loci in commercial pig populations. Our results suggest shared selection objectives and genetic architectures across swine populations. The identified polygenic selection highlights loci that are important to swine production.

**Supplementary Information:**

The online version contains supplementary material available at 10.1186/s12711-023-00836-9.

## Background

Broadly, population genetic methods are used to identify three types of directional selection in genomic data. First, hard selective sweeps are the signatures of rapid selection in which one haplotype is selected to fixation within a population. Under this rapid selection, variation surrounding the selected mutation is dragged or hitchhikes with the selected mutation resulting in large tracks of reduced nucleotide diversity and increased haplotype homozygosity. Soft selective sweeps are similar to hard sweeps in that the diversity is reduced around the selected locus, but the selected DNA variants are on more than one haplotype, via selection on standing variation, recurrent mutation or migration [1, 2]. Finally, polygenic selection leads to a large change in a phenotype that results from small changes in allele frequency at hundreds or thousands of loci [3].

Over the past 300 years, artificial selection in pigs has led to the formation of pig breeds with well-defined breed characteristics and considerable across-breed variation in phenotypes that are related to economically relevant traits [4]. Pig breeders placing selection pressure on certain qualitative phenotypes such as coat color and ear morphology and quantitative phenotypes such as feed efficiency, average daily gain, and backfat depth has left signatures of selective sweeps across the genomes of pig populations [5]. Selective sweeps are large, rapid changes in allele frequency which drag neighboring variation, leaving pronounced signatures of selection. In general, selective sweeps are associated with phenotypes that underlie the divergence of pig breeds, and have been identified in pig genomes by several studies [4–6]. However, pig breeders, are more concerned with increasing rates of genetic gain in quantitative traits [7], which are influenced by hundreds or thousands of genes. Furthermore, the selection index method has been the preferred approach to improve the aggregate genetic merit of pigs by combining data from multiple quantitative traits [8, 9], further increasing the number of genes under selection. Artificial selection using selection indices in pig breeding programs has been proven to cause significant changes in the mean phenotype of any one trait that is included in the breeding objective [10–12]. However, artificial selection pressure, especially over relatively short time scales, causes only subtle changes to allele frequencies at quantitative trait loci (QTL) across the genome [13, 14]. In addition, loci that affect traits that are not explicitly included in the selection index, such as innate immunity, have been implicated to undergo frequency changes as a result of selection pressure applied in livestock breeding programs [14, 15].

Deciphering the genetic basis of phenotypic diversity in species that are raised for meat production is a much studied research area in livestock genomics [14, 15]. Understanding selection in livestock populations is of paramount importance when evaluating the genomic basis of phenotypic variation within a genetic line, breed, or an entire livestock population over time. Identifying polygenic selection detects loci that have been subjected to consistent increases or decreases in allele frequency that are significantly larger than those due to random genetic drift [16–18]. Unlike hard or soft sweeps, polygenic selection does not leave distinctive signatures on the genome [14]. With current technologies such as single nucleotide polymorphism (SNP) arrays, temporally distributed genotypes, and increased computing resources, statistical analysis of polygenic selection is now feasible. Identification of regions of the genome that have been altered due to artificial selection pressure is highly beneficial in determining QTL that are under selection [14]. When results of selection mapping analyses are combined with results from phenotype-based genome-wide association studies (GWAS), QTL that are associated with phenotypic variation of traits within breeding objectives can be supported by multiple lines of evidence [19]. Moreover, within such selection mapping analyses, there are opportunities to evaluate results within or across genetic lines or breeds, which can highlight differences in selection objectives across livestock breeding programs. Selection mapping analyses are not limited to increasing knowledge with respect to the selection and evolution of species. Furthermore, based on the results from selection analyses, SNP assays used for the genomic prediction of breeding values in livestock populations can be refined in order to reduce extraneous statistical noise and increase prediction accuracy. This prioritization of SNPs can be accomplished by excluding SNPs that have not undergone significant changes due to directional selection or have not contributed to genetic change in traits that are included in the breeding objective.

Generation proxy selection mapping (GPSM) has been used as an analytical method for the detection of polygenic selection loci in populations [14, 15, 20]. In this approach, animal birth date (or other generation proxy) is fit as the dependent variable, and SNPs that are strongly associated with birth date are identified. If a SNP is under directional selection pressure, changes in its allele frequency will generally be consistent over time, and an animal’s genotype will be strongly associated with birth date [14, 15]. In addition, a major advantage of the GPSM methodology applied to livestock species over other methods, such as site frequency spectrum and linkage disequilibrium-based methods [21], is its ability to adjust for demography and confounding due to non-random ascertainment of genotype samples, population structure, inbreeding, or kinship with the use of a genomic relationship matrix (GRM) [14, 15]. Generation proxy selection mapping has been proven to be effective and accurate in identifying loci with allele frequency changes due to polygenic selection (as opposed to loci-specific allele frequency changes due to random genetic drift) in beef cattle populations that have been exposed to artificial selection for approximately 50 years [14]. However, there are stark differences between beef and swine breeding programs. For example, generation intervals in pigs are much shorter than in cattle (2–2.5 versus 4–5 years, respectively) [22]. Thus, for traits with similar evaluation accuracy and assuming similar selection intensity, comparable amounts of genetic gain are expected in approximately half the time for pig populations versus beef cattle populations. Moreover, due to the increasing adoption of specialized sire and dam lines, the classical “breeding pyramid”, and vertical integration in the swine industry, breeding objectives within a population of pigs tend to be more focused than breeding objectives within beef breeds, where each breeder and farm have their own breeding objectives that may be poorly defined. These differences between cattle and swine breeding programs contribute to variation in the effect of artificial selection on allele frequencies over time. The objectives of the current study were to (1) use GPSM to identify loci under artificial selection in three purebred populations and one crossbred population of pigs, and (2) compare and contrast the effect of artificial selection patterns among the genotypes of each population in the context of a swine breeding company.

## Methods

### Population background

In this study, we used four populations of pigs, using data owned by The Maschhoff’s, Limited Liability Company (LLC), Carlyle, IL, USA. Within each population, a selection index was used to identify boars and gilts with superior genetic merit to return to the breeding population at the nucleus level. Breeding population-specific selection indices for all populations included expected progeny differences (EPD) for growth and carcass traits such as increased feed efficiency and average daily gain, decreased backfat depth, and increased *longissimus* muscle area. In addition, selection indices for two of the four breeding populations (Landrace and Yorkshire) also emphasized maternal reproductive traits and included EPD for increased number and weight of piglets born and weaned.

### Pedigree and genotype data

A pedigree consisting of individual, sire, and dam identification, birth date, and genetic line for 1,247,982 pigs was provided by The Maschhoff’s, LLC. Information regarding the number of sires and dams, founder pigs, and generations within each population is summarized in Table 1. From a subset of 16,802, 19,342, 18,368, and 8532 pigs from the Duroc, Landrace, Yorkshire, and crossbred populations, respectively, genotypes were collected using a GGP Porcine 50K (Neogen, Corp., Lansing, Michigan, USA) SNP array. Genomic coordinates for each SNP were from the Sscrofa 11.1 reference genome [23]. Sample collection and subsequent genotyping were conducted on all viable male selection candidates prior to their removal from performance testing trials. In addition, all female animals selected to return to the nucleus breeding herd were genotyped. Information regarding the number of sires and dams, founder pigs, birth date ranges, and generations for genotyped pigs within each population is summarized in Table 2.

**Table 1:** Summary of pedigree records for all pigs

Population	Pigs, n	Founders, n^a^	Sires, n	Dams, n	Birth month and year		Generations, n
					Minimum	Maximum	
Duroc	114,038	742	939	6190	March 1982	September 2020	14
Landrace	236,385	1778	706	12,856	January 1993	September 2020	11
Yorkshire	207,366	730	765	10,749	June 1980	September 2020	14
Cossbred	690,193	2025	647	19,627	March 2015	August 2020	14

^a^A founder was a pig of generation 0; only the sire side of the pedigree was known for crossbred pigs

n = number

**Table 2:** Summary of pedigree records for all genotyped pigs

Population	Pigs, n	Founders, n^a^	Sires, n	Dams, n	Birth month and year		Generations, n
					Minimum	Maximum	
Duroc	16,802	17	500	3596	August 2010	April 2020	14
Landrace	19,342	82	512	5862	August 2010	April 2020	10
Yorkshire	18,368	18	446	5367	January 2011	April 2020	14
Crossbred	8532	–	206	4428	March 2015	September 2019	13

^a^A founder was a pig of generation 0; only the sire side of the pedigree was known for crossbred pigs

n = number

### Preparation of genotype data and overview of analyses

The dependent variable for all analyses was birth date (AGE) calculated as the difference, in months, between each pig’s birth month and January 2006. Pigs from the entire dataset of genotyped pigs were separated into 15 subsets based on population or combination of populations. Analyses were conducted using SNPs located on the pig autosomes only*,* i.e., chromosomes 1–18. Genotype quality control was performed using the PLINK v1.9 software [24] for each subset. SNPs with a genotype call rate lower than 0.90 or a minor allele frequency lower than 0.01 were removed from the data. In addition, individual pigs that had a genotype call rate lower than 0.90 were removed from the dataset.

The percentage of Duroc, Landrace, and Yorkshire ancestry was predicted for each pig using the fastSTRUCTURE algorithm [25], with the *K* parameter set to 3. Purebred pigs that were predicted to have a breed proportion less than 95% of their assigned genetic line (Duroc, Landrace, or Yorkshire) were removed from all subsequent analyses, as these may be due to sample swaps. While predicted breed proportions were estimated for the crossbred pigs, none were removed from the genotyped sample, as deviations from expected breed proportions cannot be distinguished from deviations due to Mendelian sampling or noise of ancestry prediction. Genomic relationship matrices (GRM) were estimated for each subset using the GCTA v1.93.2 software [26] and the method described by Yang et al. [27], and these GRM were used in all subsequent analyses. To visualize the genomic relatedness between lines, the ‘pca’ function of the GCTA software [28] was also used to conduct a principal component analysis (PCA) on a GRM for all Duroc, Landrace, Yorkshire, and crossbred pigs. A summary of the numbers of pigs and SNPs after quality control and of all subsequent analyses performed for each subset is in Table 3. Descriptive statistics of AGE by genetic line were calculated using the ‘dplyr’ package [29] in the statistical analysis software R [30]. Figures were generated using the ‘ggplot2’ [31] package of R.

**Table 3:** Summary of subsets of genotyped pigs and conducted analyses after genotype quality control

Subset	Populations	Pigs, n	SNPs, n	Analyses		
				Univariate VCE	Bivariate VCE	GWAS
1	Duroc	16,595	38,294	X		X
2	Landrace	15,457	45,085	X		X
3	Yorkshire	15,772	45,027	X		X
4	Crossbred	8447	46,529	X		X
5	Duroc and Landrace	32,066	45,999	X	X	X
6	Duroc and Yorkshire	32,387	46,106	X	X	X
7	Duroc and crossbred	25,053	46,341	X	X	X
8	Landrace and Yorkshire	31,240	46,253	X	X	X
9	Landrace and crossbred	23,905	46,440	X	X	X
10	Yorkshire and crossbred	24,230	46,449	X	X	X
11	Duroc, Landrace and Yorkshire	47,849	46,428	X		X
12	Duroc, Landrace, and crossbred	40,513	46,415	X		X
13	Duroc, Yorkshire, and crossbred	40,837	46,424	X		X
14	Landrace, Yorkshire, and crossbred	39,688	46,458	X		X
15	Duroc, Landrace, Yorkshire, and crossbred	56,296	46,456	X		X

*VCE* variance component estimation, *GWAS* genome-wide association study; n = number

Depending on data subset, certain combinations of the following three statistical analyses were performed on AGE: (1) univariate variance component estimation, (2) bivariate variance component estimation, and (3) univariate genome-wide association using a mixed linear model to estimate SNP associations.

### Univariate variance component estimation

To estimate the proportion of variance in AGE explained by genome-wide SNPs (PVE) for each data subset (Table 3), the following model was fit using the GCTA software:

$$\mathbf{y}={\mathbf{1}}\mu +\mathbf{Z}\mathbf{g}+\mathbf{e},$$

$$\mathbf{g}\sim \mathrm{N}\left({\mathbf{0}},\mathbf{G}{\upsigma }_{\mathrm{g}}^{2}\right),$$

$$\mathbf{e}\sim \mathrm{N}\left({\mathbf{0}},{\mathbf{I}\upsigma }_{\mathrm{e}}^{2}\right),$$

where $\mathbf{y}$ is the vector of observations for AGE, $\mu$ is the overall mean for AGE, $\mathbf{g}$ is the vector of random polygenic effects, $\mathbf{Z}$ is the incidence matrix relating AGE in $\mathbf{y}$ to random polygenic effects in **g**, and $\mathbf{e}$ is the vector of random residuals, $\mathbf{G}$ is the genomic relationship matrix, and $\mathbf{I}$ is an identity matrix. Additive genetic (${\sigma }_{g}^{2}$) and residual (${\sigma }_{e}^{2}$) variance components were estimated using average information restricted maximum likelihood. The PVE was then estimated as follows:

$$\mathrm{PVE}=\frac{{\upsigma }_{\mathrm{g}}^{2}}{{\upsigma }_{\mathrm{g}}^{2}+{\upsigma }_{\mathrm{e}}^{2}}.$$

### Bivariate variance component estimation

Genetic correlations (${\mathrm{r}}_{\mathrm{G}}$) between each population (Table 3) for AGE were estimated using bivariate mixed linear models, fitted in the GCTA software, of the following form:

$$\left[\begin{array}{c}{\mathbf{y}}_{\mathbf{1}}\\ {\mathbf{y}}_{\mathbf{2}}\end{array}\right]=\left[{\mathbf{1}}\right]\left[\begin{array}{c}{\mu }_{1}\\ {\mu }_{2}\end{array}\right]+\left[\begin{array}{cc}{\mathbf{Z}}_{\mathbf{1}}& \mathbf{0}\\ \mathbf{0} & {\mathbf{Z}}_{\mathbf{2}}\end{array}\right]\left[\begin{array}{c}{\mathbf{g}}_{\mathbf{1}}\\ {\mathbf{g}}_{\mathbf{2}}\end{array}\right]+\left[\begin{array}{c}{\mathbf{e}}_{\mathbf{1}}\\ {\mathbf{e}}_{\mathbf{2}}\end{array}\right],$$

where ${{\varvec{y}}}_{\mathbf{1}}$ and ${{\varvec{y}}}_{\mathbf{2}}$ are the vectors of observations for AGE for two populations 1 and 2, respectively, ${\mu }_{1}$ and ${\mu }_{2}$ are the overall means for AGE for each population, respectively, ${\mathbf{g}}_{\mathbf{1}}$ and ${\mathbf{g}}_{\mathbf{2}}$ are the vectors of random polygenic effects for each pig in the two populations, ${\mathbf{e}}_{\mathbf{1}}$ and ${\mathbf{e}}_{\mathbf{2}}$ are the vectors of the residuals for AGE of the two populations, and ${\mathbf{Z}}_{\mathbf{1}}$ and ${\mathbf{Z}}_{\mathbf{2}}$ are the incidence matrices for the random polygenic effects in ${\mathbf{g}}_{\mathbf{1}}$ and ${\mathbf{g}}_{\mathbf{2}}$, respectively. Additive genetic variance of ${\mathbf{g}}_{\mathbf{1}}$ and ${\mathbf{g}}_{\mathbf{2}}$ (${\sigma }_{g1}^{2}$ and ${\sigma }_{g2}^{2}$, respectively), additive genetic covariance between ${\mathbf{g}}_{\mathbf{1}}$ and ${\mathbf{g}}_{\mathbf{2}}$ (${\sigma }_{g1,g2}$), and residual variance of ${\mathbf{e}}_{\mathbf{1}}$ and ${\mathbf{e}}_{\mathbf{2}}$ (${\sigma }_{e1}^{2}$ and ${\sigma }_{e2}^{2}$, respectively) were estimated using average information restricted maximum likelihood with the variance–covariance matrix ($\mathbf{V}$) defined as:

$$\mathbf{V}= \left[\begin{array}{ll}{\mathbf{Z}}_{\mathbf{1}}\mathbf{G}{\mathbf{Z}}_{\mathbf{1}}^{\mathrm{^{\prime}}}{\sigma }_{{g}_{1}}^{2}+\mathbf{I}{\sigma }_{{e}_{1}}^{2}& {\mathbf{Z}}_{\mathbf{1}}\mathbf{G}{\mathbf{Z}}_{\mathbf{2}}^{\mathrm{^{\prime}}}{\sigma }_{{g}_{1}{g}_{2}}\\ {\mathbf{Z}}_{\mathbf{2}}\mathbf{G}{\mathbf{Z}}_{\mathbf{1}}^{\mathrm{^{\prime}}}{\sigma }_{{g}_{1}{g}_{2}}& {\mathbf{Z}}_{\mathbf{2}}\mathbf{G}{\mathbf{Z}}_{\mathbf{2}}^{\mathrm{^{\prime}}}{\sigma }_{{g}_{2}}^{2}+\mathbf{I}{\sigma }_{{e}_{2}}^{2}\end{array}\right],$$

where $\mathbf{G}$ and $\mathbf{I}$ are the genomic relationship and identity matrix, respectively. Genetic correlations were then estimated by GCTA using the following formula:

$${\mathrm{r}}_{\mathrm{G}}=\frac{{\upsigma }_{\mathrm{g}1,\mathrm{g}2}}{\sqrt{{\upsigma }_{\mathrm{g}1}^{2}\cdot {\upsigma }_{\mathrm{g}2}^{2}}}.$$

### Generation proxy selection mapping (GPSM)

Generation proxy selection mapping analyses were conducted to detect SNPs with changes in allele frequency over time within each subset (Table 3). To accomplish this, single-SNP univariate mixed linear models were fit in GCTA as part of the GWAS of AGE, with the models defined as follows:

$$\mathbf{y}=\mu + {\mathbf{x}}_{{\varvec{s}}}{\mathrm{b}}_{{\varvec{s}}}+\mathbf{Z}\mathbf{g}+\mathbf{e},$$

$$\mathbf{g}\sim \mathrm{N}\left({\mathbf{0}},\mathbf{G}{\upsigma }_{\mathrm{g}}^{2}\right),$$

$$\mathbf{e}\sim \mathrm{N}\left({\mathbf{0}},{\mathbf{I}\upsigma }_{\mathrm{e}}^{2}\right),$$

where $\mathbf{y}$ is a vector of pig’s generation proxy (AGE), $\mu$ is the mean AGE, ${\mathbf{x}}_{{\varvec{s}}}$ is the vector of SNP genotypes for each pig at SNP *s*, and ${\mathrm{b}}_{{\varvec{s}}}$ is the SNP effect for SNP *s*. Confounding due to population structure, relatedness, and inbreeding are controlled by the random polygenic terms in the vector $\mathbf{g}$, and $\mathbf{Z}$ is the incidence matrix for the effects in $\mathbf{g}$. In addition, $\mathbf{G}$ is the genomic relationship matrix, and $\mathbf{I}$ is an identity matrix. Additive genetic (${\sigma }_{g}^{2}$) and residual (${\sigma }_{e}^{2}$) variance components were estimated using average information restricted maximum likelihood. However, these variance components were not of interest as a part of the GPSM analyses as they were estimated previously as a part of the univariate variance component estimation analysis. The genomic relationship matrix accounts for the relationships within and across populations, thus preventing spurious associations due to population structure when we analyzed combination of populations in different subsets. *P*-values of the estimated SNP effects were converted to false discovery rate (FDR) corrected *Q*-values using the ‘qvalue’ package [32] of R, and a significance threshold of *Q* < 0.10 was used for all analyses.

### Variance component and GPSM analyses using simulated data

Variance component and GPSM analyses of purebred pigs (subsets 1, 2, 3, 5, 6, 8, and 11; Table 3) were conducted using gene-drop simulated genotype data produced by using the ‘AlphaSimR’ package [33, 34] using the pedigree data of the analyzed pigs. The objective of these gene-drop analyses was to ensure that the GPSM results obtained from real data were due to artificial selection as opposed to random genetic drift. For the univariate variance component estimation and the GPSM analyses for each of the subsets 1, 2, 3, 5, 6, 8, and 11 (Table 3), 5000 founder pig haplotypes were simulated using AlphaSimR’s MaCS [35] wrapper, with the demography parameter set to “GENERIC”. Each of the simulated haplotypes contained 90,000 segregating sites that were evenly located along the 18 autosomes. Then, using the pedigreeCross function [33], founder pigs in the pedigree of each subset were assigned genotypes at random from the simulated population of 5000 pigs. Simulated founder pig haplotypes were then dropped through each pedigree to simulate the exact matings that have occurred in The Maschhoff’s breeding program (each allele inherited by progeny was randomly assigned according to recombination and segregation). Lastly, pigs with genotypes that were used in the real analyses were extracted from each subset along with a “SNP array” of randomly selected loci equivalent to the number of SNPs used in the real analyses (Table 3). Univariate variance component estimation and GPSM analyses were conducted using the same statistical models and software, the simulated genotypes, and the AGE values from the real analyses. For the Duroc, Landrace, and Yorkshire populations, the above process was replicated five times to ensure that the results from the analysis of simulated data were not affected by randomness within the simulation process.

In the bivariate variance component analyses on simulated data (subsets 5, 6, and 8; Table 3), founder pig haplotypes were simulated in two different ways. First, founder pig haplotypes were simulated as one group that consisted of 15,000 founder pigs (Method 1). The objective of this method was to simulate a scenario where each combination of populations had recently diverged; thus, the founder animals for each population have the same genotypes. For the second method, founder pig haplotypes were simulated separately for each population, the random number generator in R was changed between each simulation, and then the two founder pig haplotypes were combined (Method 2). Using Method 2, the simulated genotypes differed considerably between founder pigs in each population combination, which represented pairs of populations that were completely unrelated. These two strategies represent the extremes of coalescent times between breeds, rather than assuming a specific number of generations since the divergence of the breeds. Samples of pigs with simulated genotypes were created in the same manner as described above for the univariate analyses. Bivariate variance component analyses were then conducted using both samples of simulated genotypes from each method and each pairwise comparison of subsets 5, 6, and 8. Results from all the analyses using simulated data and those using real data were then compared in a one-to-one fashion.

### Investigation of GPSM associations

The number of shared significant GPSM associations between and across each purebred population and the crossbred population (subsets 1–4; Table 3) were visualized using the R package ‘UpSetR’ [36]. The ‘GALLO’ package of R [37] was used to identify positional candidate genes (file Sus_scrofa.Sscrofa11.1.105.gtf.gz downloaded from the ‘Pig’ section of Ensembl [38]) and quantitative trait loci (file Animal_QTLdb_release76_pigSS11.gff.gz downloaded from the ‘PigQTLdb’ section of AnimalQTLdb [39]) within 100 kb upstream and downstream of each significant SNP identified by GPSM in the Duroc, Landrace, Yorkshire, and crossbred populations (subsets 1 to 4; Table 3). In addition, the ‘gwascat’ package of R [40] was used to download the most recent version of the NHGRI-EBI GWAS catalog [41]. The traits from the NHGRI-EBI GWAS catalog that were associated with the genes annotated by ‘GALLO’ were identified and are discussed in this paper.

## Results

### Descriptive statistics and principal components analysis

**Fig. 1:**
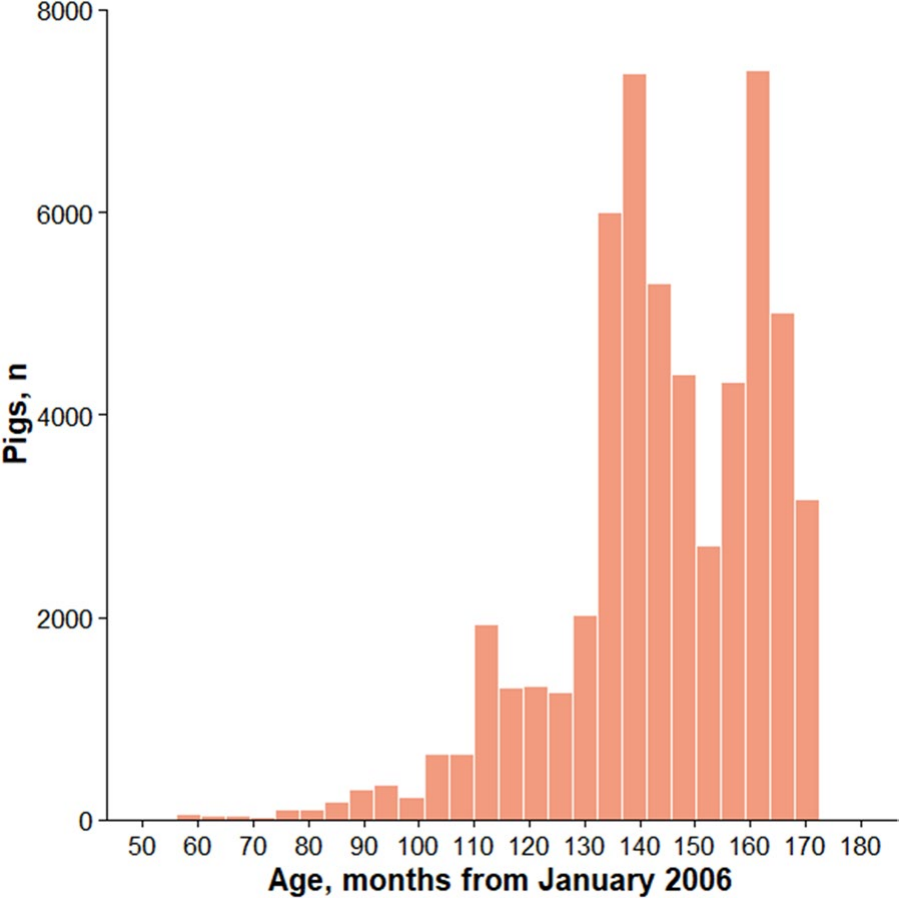
Distribution of AGE for all genotyped pigs. For each pig, AGE was calculated as the number of months between each pig’s birth month and January 2006. A pig with a negative, zero, or positive AGE was born before January 2006, during January 2006, or after January 2006, respectively

**Fig. 2:**
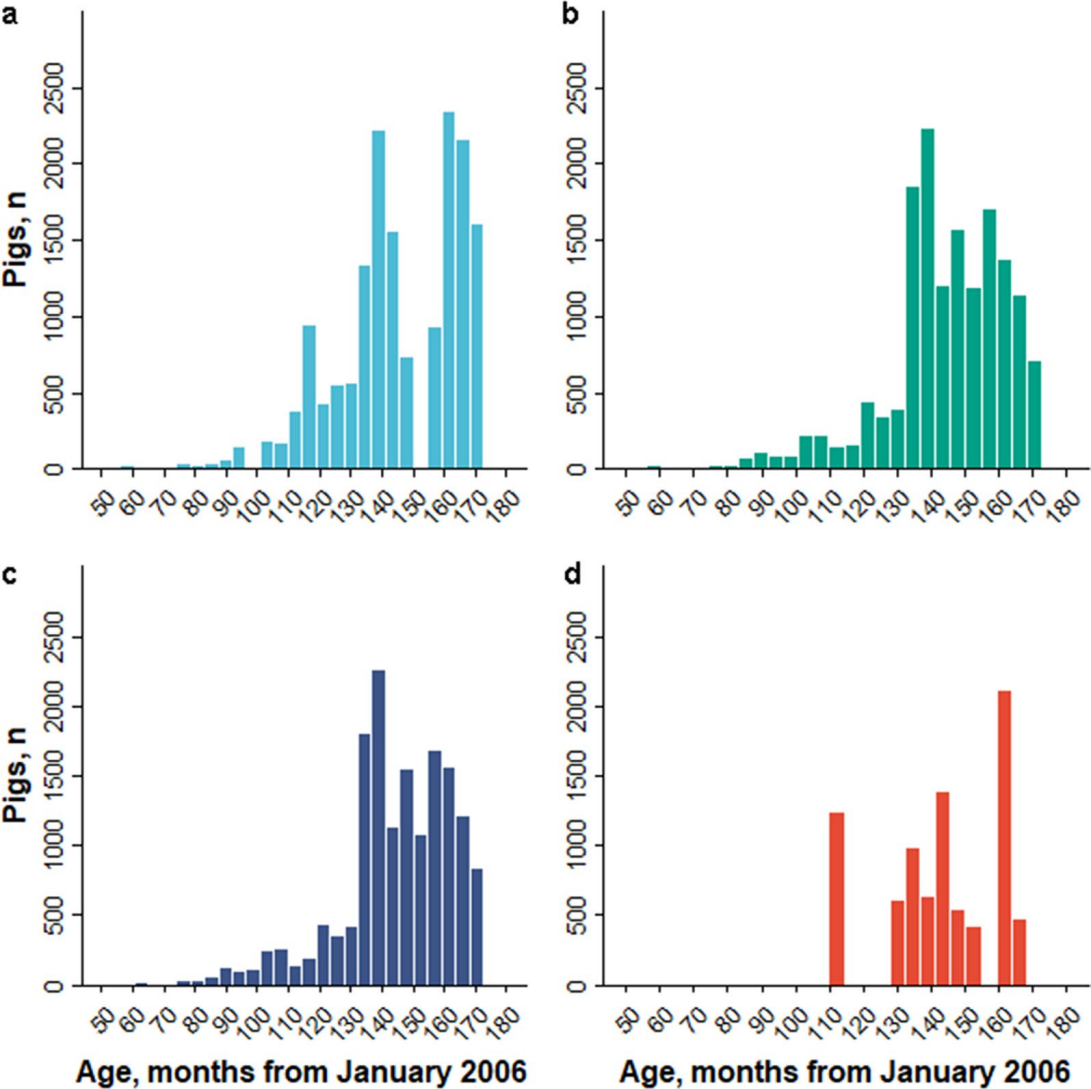
Distributions of AGE for genotyped pigs in Duroc, Landrace, Yorkshire and crossbreed lines. For each Duroc (**a**), Landrace (**b**), Yorkshire (**c**), and crossbred (**d**) pig, AGE was calculated as the number of months between each pig’s birth month and January 2006. For example, a pig with an age of 120 was born in January 2016

Descriptive statistics of AGE for each subset are in Table 4. In addition, the raw distributions of AGE are shown in Fig. 1 for all pigs (subset 15; Table 3) and in Fig. 2 for subsets 1 through 4 (Table 3). The histograms of AGE depict the frequency of genotype sampling across all populations and within each population for the duration of The Maschhoff’s breeding program (Figs. 1, 2, respectively). In general, descriptive statistics for AGE were similar across each subset (Table 4). However, the range and standard deviation of AGE for the crossbred pigs were smaller than those for the other subsets, as genotyping of these pigs did not begin until March 2015 (Table 2). Thus, the number of genotyped crossbred pigs was approximately half the number of Duroc, Landrace, and Yorkshire pigs. Furthermore, the histograms of AGE for each subset were left-skewed, indicating that the number of pigs genotyped per year in each subset generally increased from the start of The Maschhoff’s SNP collection platform from 2010 until 2020.

**Table 4:** Descriptive statistics by subset for AGE (difference, in months, between each pig’s birth date and January 2006)

Subset	Populations	Pigs, n	Mean	SD	Minimum	Maximum
1	Duroc	16,595	144.9	20.44	55	171
2	Landrace	15,457	144.0	18.23	55	171
3	Yorkshire	15,772	144.2	18.46	64	171
4	Crossbred	8447	142.8	17.20	110	164
5	Duroc and Landrace	32,066	144.5	19.41	55	171
6	Duroc and Yorkshire	32,387	144.6	19.50	55	171
7	Duroc and crossbred	25,053	144.2	19.43	55	171
8	Landrace and Yorkshire	31,240	144.1	18.34	55	171
9	Landrace and crossbred	23,905	143.6	17.88	55	171
10	Yorkshire and crossbred	24,230	143.7	18.04	64	171
11	Duroc, Landrace and Yorkshire	47,849	144.4	19.10	55	171
12	Duroc, Landrace, and crossbred	40,513	144.1	18.98	55	171
13	Duroc, Yorkshire, and crossbred	40,837	144.2	19.06	55	171
14	Landrace, Yorkshire, and crossbred	39,688	143.8	18.11	55	171
15	Duroc, Landrace, Yorkshire, and crossbred	56,296	144.2	18.84	55	171

*SD* standard deviation; n = number

**Fig. 3:**
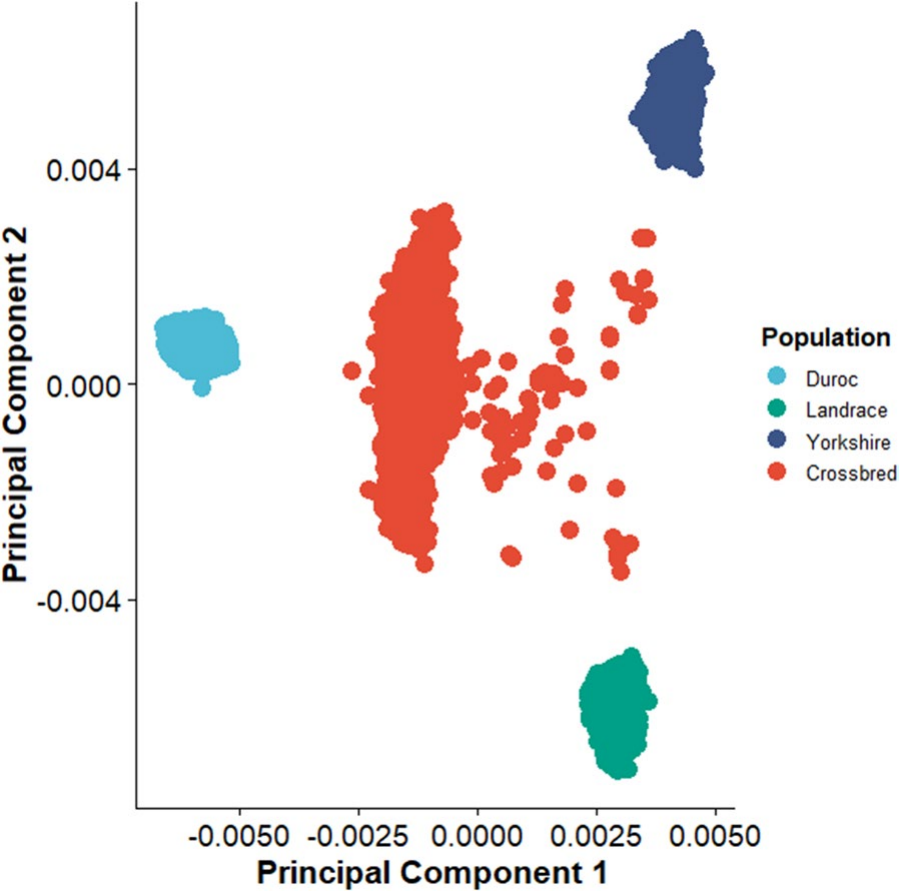
Principal component analysis of GRM that included analyzed genotyped pigs. Four clusters appear in the scatterplot. Individual pigs within each colored cluster constitute a population. Populations of pigs that are located closer to each other are genetically more similar

The results from the PCA of the GRM that included analyzed pigs from each population (subset 15; Table 3) are presented in Fig. 3. By plotting principal component 1 versus principal component 2 for the genomic relatedness of these four populations, four defined clusters were visualized, as expected. In addition, the cluster for the crossbred population was located halfway between the Duroc population cluster and the Landrace and Yorkshire population clusters along principal component 1 and halfway between the Landrace and Yorkshire population clusters along principal component 2 (Fig. 3). McVean et al. [42] postulated that the location of an admixed population of individuals on a PCA plot relative to its source populations relates directly to the admixture proportion of these individuals among the source populations. Thus, the location of the crossbred cluster in Fig. 3 confirms approximately a 50/25/25 admixture among the Duroc, Landrace, and Yorkshire populations, respectively. This result was expected given the design of The Maschhoff’s mating program for their commercial test herd, which mates Duroc sires to Landrace × Yorkshire dams.

### Univariate and bivariate variance component estimation

The proportion of variation explained by genome-wide SNPs of the dependent variable AGE for all 15 subsets is in Table 5. These values ranged from 0.81 to 0.94 (Table 5) and were significantly greater than 0 (*P* < 0.001) using the likelihood ratio test. Previous GPSM simulations have shown that GPSM PVE is indicative of population demographic history [14]. Given that the descriptive statistics and distributions of AGE were generally similar across subsets (Table 4; Figs. 1, 2), these PVE results suggest that the four populations have similar demographic characteristics, such as inbreeding, effective population sizes, and pedigree structure. The results of the univariate variance component estimation in subsets containing purebred populations (subsets 1, 2, 3, 5, 6, 8, and 11; Table 3) using simulated genotypes are in Table 6. Estimated PVE for subsets with simulated genotypes were generally similar to those obtained with real data, ranging from 0.83 to 0.93 (Table 6), and were significantly different from 0 (*P* < 0.001) based on the likelihood ratio test. Between the univariate variance component estimation analyses using real and simulated genotype data, the pedigree structure, AGE values, and number of SNPs were the same for each subset; however, the genomic relationship between each pairwise combination of pigs was different. For the Landrace and Yorkshire populations, the real PVE was higher than the simulated PVE by only 0.02 and 0.01, respectively. However, for the Duroc population, the real PVE was 0.11 higher than the simulated PVE. In addition, PVE from the univariate variance component analyses were similar across replicates of simulated genotype data. For example, PVE ranged from 0.82 to 0.84 (mean of 0.83 ± 0.004), 0.85 to 0.87 (mean of 0.86 ± 0.003), and 0.84 to 0.85 (mean of 0.842 ± 0.0020) for the Duroc, Landrace, and Yorkshire populations, respectively, across five replications of simulated genotype data per population (see Additional file 1: Table S1). Thus, the variance components and PVE are not impacted by the stochastic generation of the simulated genotypes.

**Table 5:** Proportion of variation in AGE (difference, in months, between each pig’s birth date and January 2006) explained by SNPs for each subset

Subset	Populations	Pigs, n	SNPs, n	PVE	SE
1	Duroc	16,595	38,294	0.94	0.002
2	Landrace	15,457	45,085	0.87	0.004
3	Yorkshire	15,772	45,027	0.86	0.004
4	Crossbred	8447	46,529	0.94	0.004
5	Duroc and Landrace	32,066	45,999	0.89	0.001
6	Duroc and Yorkshire	32,387	46,106	0.84	0.001
7	Duroc and crossbred	25,053	46,341	0.91	0.002
8	Landrace and Yorkshire	31,240	46,253	0.89	0.002
9	Landrace and crossbred	23,905	46,440	0.88	0.003
10	Yorkshire and crossbred	24,230	46,449	0.87	0.003
11	Duroc, Landrace and Yorkshire	47,849	46,428	0.82	0.001
12	Duroc, Landrace, and crossbred	40,513	46,415	0.84	0.001
13	Duroc, Yorkshire, and crossbred	40,837	46,424	0.81	0.001
14	Landrace, Yorkshire, and crossbred	39,688	46,458	0.87	0.001
15	Duroc, Landrace, Yorkshire, and crossbred	56,296	46,456	0.84	0.001

*PVE* proportion of variation in AGE explained by SNPs (i.e., SNP heritability); *SE* standard error; n = number

**Table 6:** Proportion of variation in AGE (difference, in months, between each pig’s birth date and January 2006) explained by SNPs for each purebred subset using simulated data

Subset	Populations	Pigs, n	SNPs, n	PVE	SE
1	Duroc	16,595	38,286	0.83	0.005
2	Landrace	15,457	45,090	0.85	0.004
3	Yorkshire	15,772	45,036	0.85	0.005
5	Duroc and Landrace (Method 1)	32,066	46,008	0.90	0.003
5	Duroc and Landrace (Method 2)	32,066	46,008	0.88	0.003
6	Duroc and Yorkshire (Method 1)	32,387	46,098	0.89	0.003
6	Duroc and Yorkshire (Method 2)	32,387	46,098	0.88	0.003
8	Landrace and Yorkshire (Method 1)	31,240	46,260	0.91	0.002
8	Landrace and Yorkshire (Method 2)	31,240	46,260	0.89	0.003
11	Duroc, Landrace, and Yorkshire (Method 1)	47,849	46,422	0.93	0.002
11	Duroc, Landrace, and Yorkshire (Method 2)	47,849	46,422	0.90	0.002

*PVE* proportion of variation in AGE explained by SNPs (i.e., SNP heritability); *SE* standard error; n = number

Method 1 = genotypes simulated as if populations recently diverged (same founder population); Method 2 = genotypes simulated as if populations are completely unrelated (different founder populations)

Genetic correlations between AGE for all pairwise combinations of the Duroc, Landrace, Yorkshire, and crossbred populations (subsets 1 through 4; Table 3) are in Table 7. In general, the genetic correlations of AGE between purebred populations were stronger than those between each purebred population and the crossbred population (Table 7). Each genetic correlation was significantly different from 0 (*P* < 0.001) based on the likelihood ratio test. Within the purebred subsets (5, 6, and 8; Table 3), the genetic correlation between Landrace and Yorkshire pigs was higher than the genetic correlation between Duroc and Landrace or Duroc and Yorkshire pigs (Table 7). This indicates that the demographic and selection histories (associations with AGE) are more similar between Landrace and Yorkshire pigs than between either of the two maternal breeds and the Duroc population. This result was expected, since Landrace and Yorkshire pigs are both selected for maternal traits while Duroc pigs are selected for increased efficiency in terminal traits. Among each pairwise combination between the crossbred population and each purebred population (subsets 7, 9, and 10; Table 3), genetic correlations for AGE were highest between the Duroc and crossbred population and were similar between crossbred and Landrace or Yorkshire pigs (Table 7). Given that Duroc pigs contribute more genetic material to the crossbred pigs than the Landrace and Yorkshire pigs, this result was expected.

**Table 7:** Genetic correlations for AGE (difference, in months, between each pig’s birth date and January 2006) between each pairwise combination of populations 1 through 4

Subset	Populations	Pigs, n	SNPs, n	r_G_	SE
5	Duroc and Landrace	32,066	45,999	0.64	0.004
6	Duroc and Yorkshire	32,387	46,106	0.67	0.023
7	Duroc and crossbred	25,053	46,341	0.50	0.018
8	Landrace and Yorkshire	31,240	46,253	0.80	0.017
9	Landrace and crossbred	23,905	46,440	0.38	0.021
10	Yorkshire and crossbred	24,230	46,449	0.43	0.020

r_G_ = genetic correlation; SE = standard error; n = number

Table 8 presents the genetic correlations from the bivariate variance component estimation analyses using simulated data. The genetic correlations of AGE between each population (subsets 5, 6, and 8; Table 3), using both methods, were not significantly different from 0 (*P* > 0.05) based on the likelihood ratio test (Table 8). This result suggests that in the absence of artificial selection pressure on economically relevant traits in each population, transmission of genotypes between generations is independent across breeds, hence the genetic correlation is expectedly zero. Moreover, negligible genetic correlations were observed across the two methods used to simulate founder populations; therefore, the length of time from population divergence likely has no effect on the genetic correlations in the presence of genetic drift. Thus, the results from this bivariate variance component analysis using simulated founder genotypes strengthen the validity of the assumptions presented above based on real genotype data in genetic lines exposed to artificial selection pressure.

**Table 8:** Genetic correlations for AGE (difference, in months, between each pig’s birth date and January 2006) between each pairwise combination of populations 1 through 3 using simulated genotype data

Subgroup	Genetic lines	Pigs, n	SNPs, n	r_G_	SE
5	Duroc and Landrace (Method 1)^2^	32,066	46,008	-0.03	0.028
5	Duroc and Landrace (Method 2)	32,066	46,008	-0.02	0.058
6	Duroc and Yorkshire (Method 1)	32,387	46,098	0.06	0.029
6	Duroc and Yorkshire (Method 2)	32,387	46,098	0.03	0.057
8	Landrace and Yorkshire (Method 1)	31,240	46,260	-0.02	0.028
8	Landrace and Yorkshire (Method 2)	31,240	46,260	-0.01	0.056

r_G_ = genetic correlation; SE = standard error; n = number

Method 1 = genotypes simulated as if populations recently diverged (same founder population); Method 2 = genotypes simulated as if populations diverged several years ago (different founder populations)

### Detection of polygenic selection with generation proxy selection mapping

**Fig. 4:**
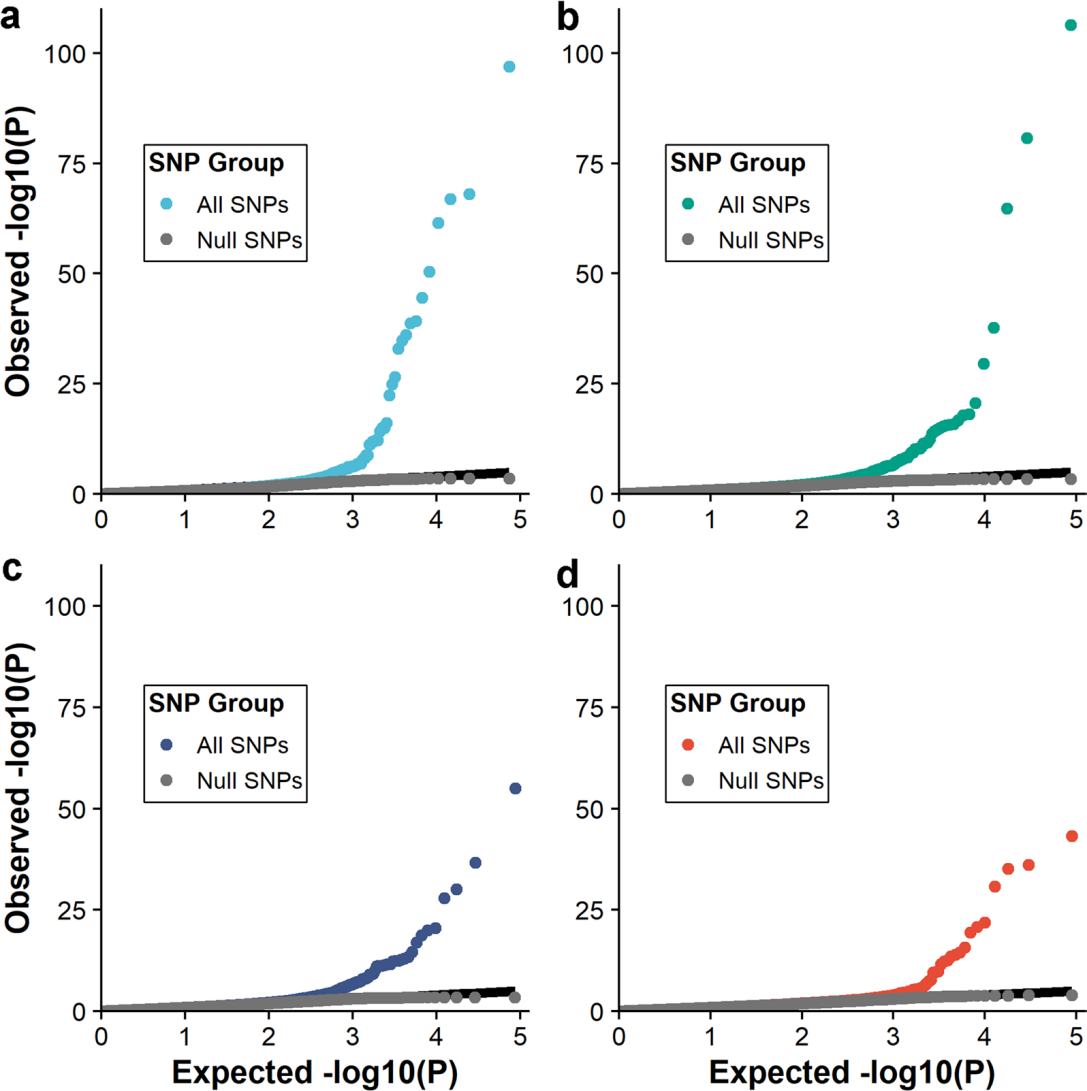
Q–Q plots for GPSM P-values from genome-wide association analyses of SNP genotype on AGE. Null SNPs (non-significant) closely followed a uniform distribution, while GPSM significant SNPs deviated from the expected uniform distribution for Duroc (**a**), Landrace (**b**), Yorkshire (**c**), and crossbred (**d**)

The number of significant SNPs (*Q* < 0.10) associated with AGE for each subset is in Table 9. Although the distribution of AGE for each subset was left-skewed and non-normal (Table 4; Figs. 1, 2), the GPSM *P*-values for independent SNP genotype association tests with AGE were well calibrated (Fig. 4). For example, *P*-values for null SNPs, which were deemed non-significant by GPSM, closely followed the expected uniform distribution, while SNPs that were significantly associated with AGE deviated from this expectation (Fig. 4). This result suggests that departures from normality in the dependent variable in a GPSM analysis does not produce spurious associations between AGE and genotype. Generation proxy selection mapping identified 49 to 854 significant SNPs (Table 9) depending on the subset. The number of significant associations generally increased as the number of samples in the subset increased, as expected, due to increased power of the GWAS.

**Table 9:** Number of SNPs significantly associated with AGE (difference, in months, between each pig’s birth date and January 2006) for each subset

Subset	Populations	Pigs, n	SNPs, n	Significant SNPs, n^a^
1	Duroc	16,595	38,294	100
2	Landrace	15,457	45,085	147
3	Yorkshire	15,772	45,027	138
4	Crossbred	8447	46,529	49
5	Duroc and Landrace	32,066	45,999	371
6	Duroc and Yorkshire	32,387	46,106	527
7	Duroc and crossbred	25,053	46,341	148
8	Landrace and Yorkshire	31,240	46,253	177
9	Landrace and crossbred	23,905	46,440	172
10	Yorkshire and crossbred	24,230	46,449	182
11	Duroc, Landrace and Yorkshire	47,849	46,428	702
12	Duroc, Landrace, and crossbred	40,513	46,415	533
13	Duroc, Yorkshire, and crossbred	40,837	46,424	609
14	Landrace, Yorkshire, and crossbred	39,688	46,458	274
15	Duroc, Landrace, Yorkshire, and crossbred	56,296	46,456	854

^a^*Q* < 0.10

n = number

One hundred, 147, 138, and 49 significant SNPs were identified by GPSM representing 0.26, 0.33, 0.31, and 0.11% of the total number of autosomal SNPs for the Duroc, Landrace, Yorkshire, and crossbred populations, respectively (subsets 1 through 4; Table 9). However, when all purebred pigs were combined into a single subset (subset 11; Table 3), GPSM identified 702 significant associations (1.51% of autosomal loci; Table 9). Moreover, the addition of crossbred pigs to subset 11, which created subset 15 (Table 3), allowed GPSM to identify 854 significant associations (1.84% of the autosomal loci; Table 9). As mentioned above, the efficacy of GPSM analyses depends on the power of the genome-wide association analyses. Thus, as more samples of SNP genotype information on a particular population of pigs are accumulated, more SNP genotypes that are associated with AGE can be detected using the GPSM method.

**Fig. 5:**
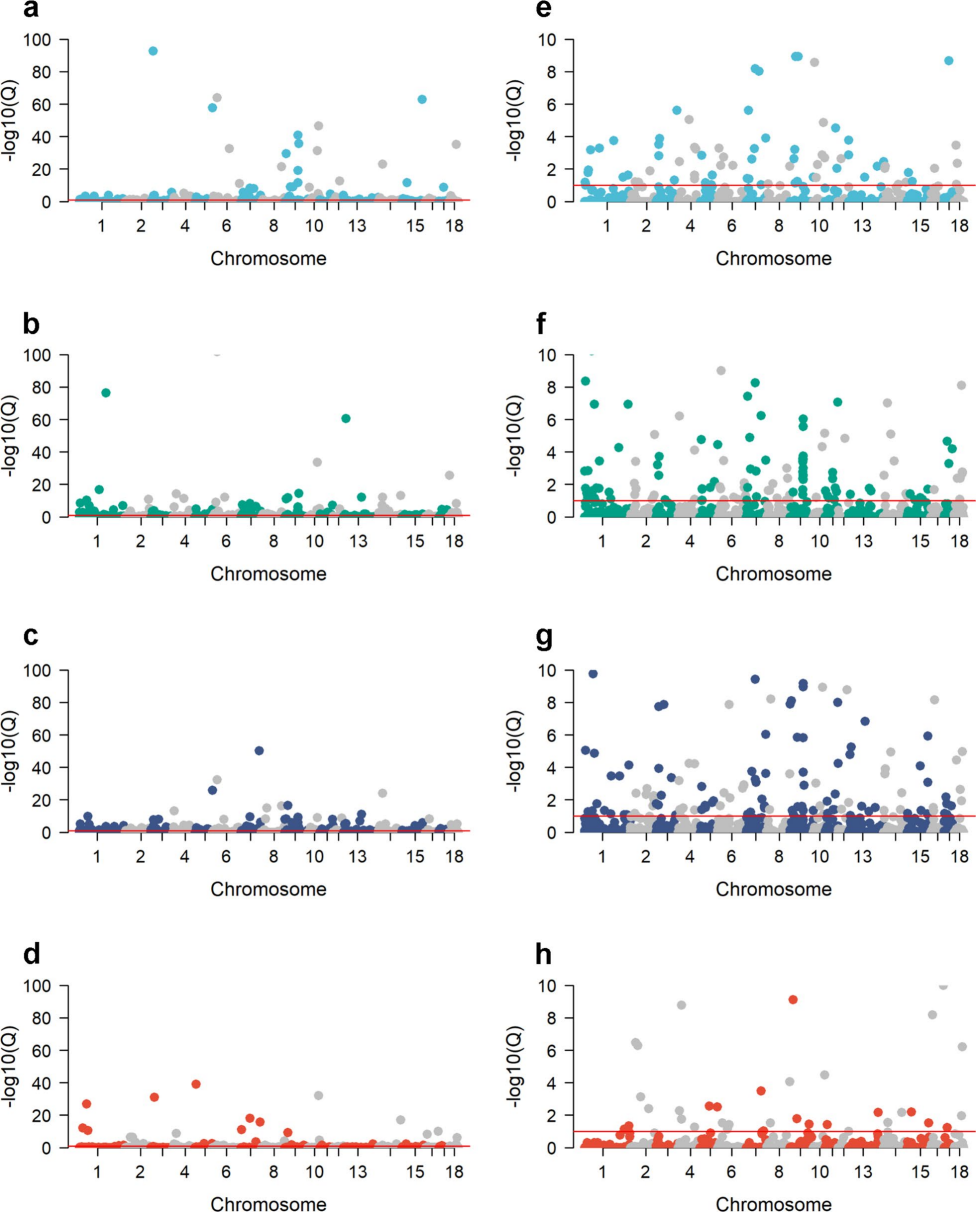
Manhattan plots of GPSM Q-values for the association between SNP genotype and AGE. Significant GPSM SNPs were detected on each chromosome, and -log_10_(*Q*-values) are shown on the Manhattan plots with full Y-axes for Duroc (**a**), Landrace (**b**), Yorkshire (**c**), and crossbred (**d**). Truncated Y-axes from 0 to 10 -log_10_(*Q*-values) reveal the polygenic nature of the selection in Duroc (**e**), Landrace (**f**), Yorkshire (**g**), and crossbred pigs (**h**)

Manhattan plots of ${-log}_{10}(Q)$ values for the associations between SNP genotypes and AGE in the Duroc, Landrace, Yorkshire, and crossbred populations (subsets 1 through 4; Table 3) are presented in Fig. 5. For each population, a plot is presented with a full (Fig. 5a–d) and a truncated Y-axis, with ${-log}_{10}(Q)$ values ranging from 0 to 10 (Fig. 5e–h). Within each subset, several significant associations between SNP genotype and AGE were identified on each chromosome by GPSM (Fig. 5). When the Manhattan plots for each genetic line have a truncated Y-axis, the genome-wide nature of the significant associations becomes more pronounced (Fig. 5e–h).

**Fig. 6:**
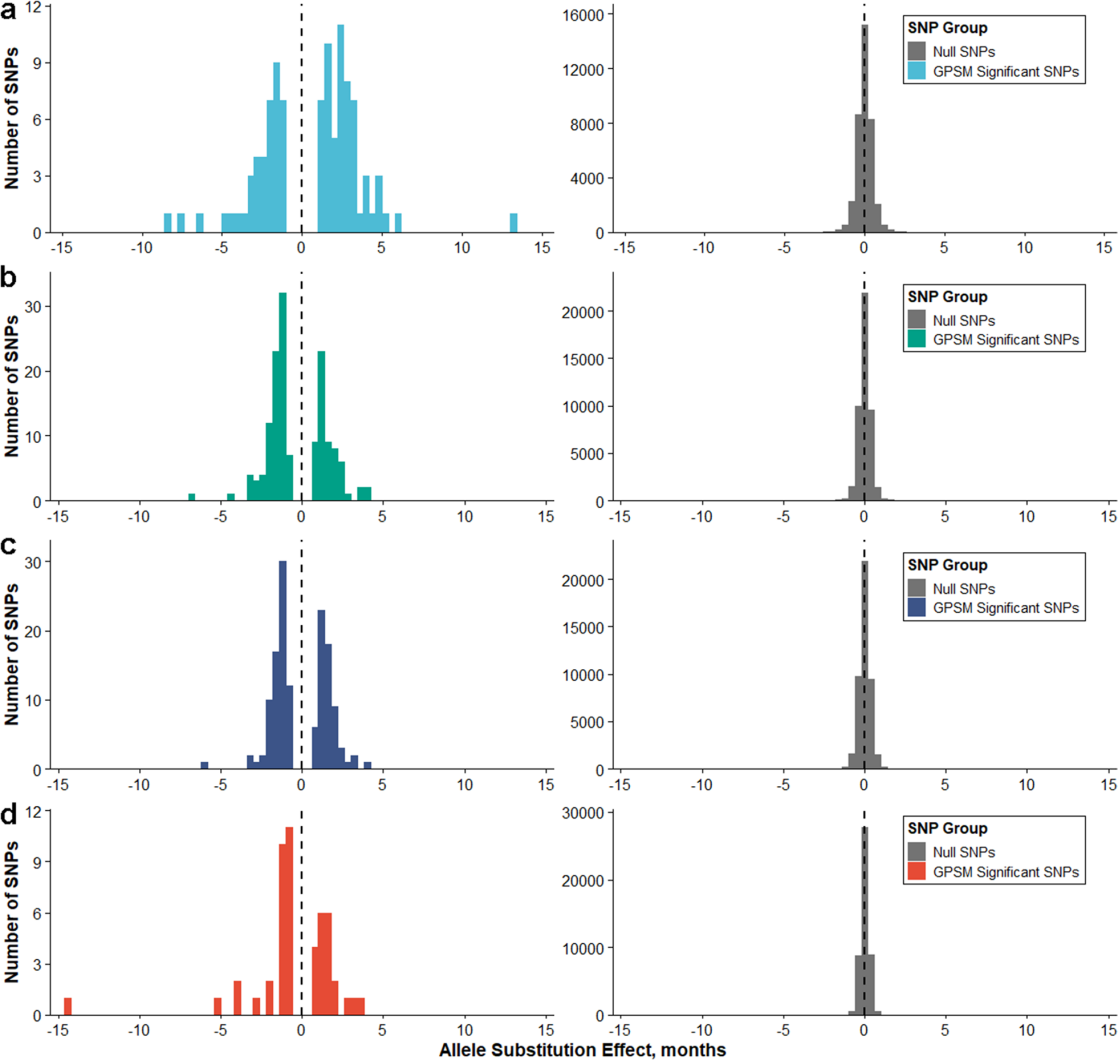
Distribution of SNP effects for null and GPSM significant markers. For Duroc (**a**), Landrace (**b**), Yorkshire (**c**), and crossbred (**d**) pigs, null SNPs (non-significant) were normally distributed with a mean near zero, while GPSM significant SNPs followed a bimodal distribution with central values for each peak located above and below zero

The distributions of SNP effects are plotted in Fig. 6. The SNP effects in each population that were significantly different from 0 were converted to absolute values to interpret differences in magnitude of this parameter across populations. Duroc pigs had the highest mean absolute value of age SNP effects for significant SNPs (2.70 months) and mean absolute values of SNP effects in significant SNPs were similar between the Landrace (1.66 months), Yorkshire (1.55 months), and crossbred (1.80 months) populations. The range in absolute values of SNP effects of GPSM significant SNPs was considerable, depending on the population. For example, in the Duroc and crossbred populations, these ranges were from 1.00 to 13.32 months and 0.70 to 14.39 months, respectively. However, for the Landrace and Yorkshire populations, these ranges were narrower (from 0.71 to 6.64 and 0.71 to 6.00 months, respectively) but were similar between the two populations. In addition, the mean change in allele frequency per year of significant SNPs for each population was 0.018 per year for Duroc (range from 0.00001 to 0.109), 0.019 per year for Landrace (range from 0.0001 to 0.082), 0.019 per year for Yorkshire (range from 0.0006 to 0.101), and 0.024 per year for crossbred (range from 0.0007 to 0.086).

The results from the GPSM analyses using randomly simulated founder genotypes are in Table 10. Out of the 11 GPSM runs on the simulated data, GPSM falsely identified significant associations with AGE in seven analyses (Table 10). However, in these analyses, a very small number of spurious associations were detected (Table 10), corresponding to error rates ranging from 0 to 0.0152% (Table 10), which are negligible. Moreover, the false positive rate for GPSM associations was stable across five replicates of simulated genotype data for the Duroc, Landrace, and Yorkshire populations (mean of 0.0031 ± 0.00192%, 0.0022 ± 0.00121%, and 0.0004 ± 0.00044%, respectively; See Additional file 2: Table S2).

**Table 10:** Number of SNPs significantly associated with AGE (difference, in months, between each pig’s birth date and January 2006) for each subset using randomly simulated genotype data

Subset	Populations	Pigs, n	SNPs, n	Significant SNPs, n^a^	Error rate, %
1	Duroc	16,595	38,286	1	0.0026
2	Landrace	15,457	45,090	0	0.0000
3	Yorkshire	15,772	45,036	2	0.0044
5	Duroc and Landrace (Method 1)	32,066	46,008	0	0.0000
5	Duroc and Landrace (Method 2)	32,066	46,008	4	0.0087
6	Duroc and Yorkshire (Method 1)	32,387	46,098	0	0.0000
6	Duroc and Yorkshire (Method 2)	32,387	46,098	7	0.0152
8	Landrace and Yorkshire (Method 1)	31,240	46,260	1	0.0022
8	Landrace and Yorkshire (Method 2)	31,240	46,260	3	0.0065
11	Duroc, Landrace, and Yorkshire (Method 1)	47,849	46,422	0	0.0000
11	Duroc, Landrace, and Yorkshire (Method 2)	47,849	46,422	6	0.0129

^a^*Q* < 0.10

Method 1 = genotypes simulated as if populations recently diverged (same founder population); Method 2 = genotypes simulated as if populations are completely unrelated (different founder populations)

Error rate = (Significant SNPs, n/SNPs, n) × 100; n = number

Analyses using Method 2 to simulate founder genotypes, which simulated completely different founder genotypes for each population, had a significantly larger number of spurious associations than those using Method 1, which simulated a single founder population for all three purebred populations (paired t-test, t = − 4.2748, df = 3, *P*-value = 0.0235). However, in commercial pig populations, divergence likely occurred in a scenario that resembles a blending of Methods 1 and 2; thus, the higher error rate in Method 2 could be inflated compared to reality in the swine industry. Nevertheless, these results suggest that GPSM is robust to allele frequency changes due to genetic drift over time. Furthermore, simulation results show that the genomic relationship matrix appropriately accounts for population stratification (combining populations in analyses), preventing spurious associations.

**Fig. 7:**
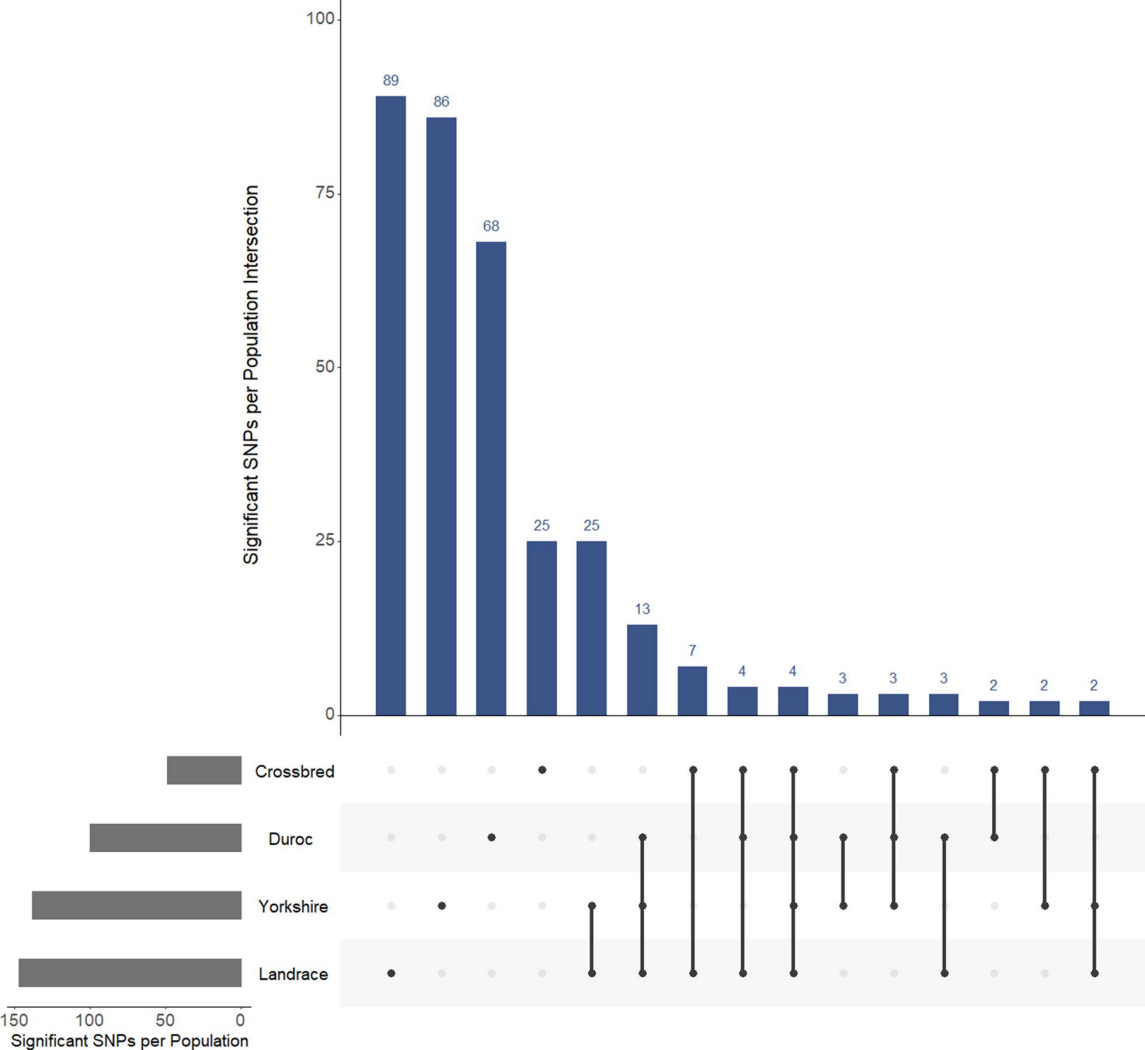
Upset plot showing the number of GPSM significant SNPs across populations. Each vertical blue bar shows the number of GPSM significant SNPs that are unique to a single population (25–89 SNPs), unique across two populations (2–25 SNPs), unique across three populations (2–13 SNPs), or unique across all four populations (4 SNPs). Horizontal gray bars present the number of GPSM significant SNPs for each genetic line

The number of shared significant GPSM SNPs across subsets 1 through 4 is presented in Fig. 7. Forty-two, 22, and four SNPs significantly associated with AGE were shared across at least two, three, or four populations, respectively (Fig. 7). Twenty-five GPSM associations were shared between the Landrace and Yorkshire populations, which was considerably more than the number of shared GPSM associations identified between all other pairwise combinations of populations (Fig. 7). In addition, 13 GPSM associations were shared across all three purebred populations. However, only two to four GPSM SNPs were unique to subsets of three populations in which crossbred pigs were included (subsets 12 through 14; Fig. 7). Top SNP associations with AGE in the Duroc, Landrace, Yorkshire, and crossbred populations are in Table 11. In general, most of the significant SNPs with the 10 largest absolute values for SNP effects were significant in at least one other subset (Table 11). In the crossbred population, only two of the top 10 large effect SNPs were unique to crossbred pigs (3, 4, and 1 out of 10 were significant across at least 2, 3 and 4 subsets, respectively; Table 11). In addition, certain SNPs exhibited large effects across multiple subsets. For example, GPSM estimated an effect for SNP 39502 of 6.19, -6.64, and 4.03 months (Table 11) in the Duroc, Landrace, and Yorkshire populations, respectively, which were 11.9, 18.4, and 10.9 SD above, below, and above the mean SNP effect within each population, respectively.

**Table 11:** Ten SNPs significantly associated with AGE (difference, in months, between each pig’s birth date and January 2006) with the largest absolute values for SNP effects within the Duroc, Landrace, Yorkshire, and crossbred populations

Subset	Populations	SNP identifier^a^	MAF	SNP effect	SE	*Q*-value	Number of subsets in which SNP was significant
1	Duroc	3420	0.24	13.32	0.310	1.49E−102	3
		41,017	0.02	8.21	0.648	3.54E−33	1
		30,819	0.07	7.71	0.368	2.05E−93	2
		35,689	0.09	6.49	0.390	2.34E−58	3
		39,502	0.47	6.19	0.353	1.24E−64	3
		18,513	0.02	5.16	0.643	2.28E−12	1
		30,855	0.13	4.87	1.025	1.56E−03	1
		49,794	0.38	4.79	0.340	1.56E−41	3
		31,005	0.11	4.66	0.880	1.31E−04	1
		6055	0.08	4.60	0.370	5.40E−32	3
2	Landrace	39,502	0.30	6.64	0.302	1.76E−102	3
		14,465	0.02	4.40	0.518	9.00E−14	3
		1063	0.46	3.95	0.207	3.69E−77	1
		22,747	0.31	3.81	0.333	2.43E−26	1
		10,745	0.42	3.52	0.206	2.66E−61	2
		38,925	0.04	3.51	0.840	1.55E−02	1
		6055	0.18	3.08	0.238	2.03E−34	3
		485	0.23	3.08	0.325	1.71E−17	2
		39,264	0.28	3.03	0.418	9.58E−10	1
		8174	0.29	3.00	0.456	8.35E−08	2
3	Yorkshire	45,804	0.02	6.00	0.664	1.14E−15	1
		39,502	0.36	4.03	0.316	4.80E−33	3
		19,756	0.17	3.40	0.486	7.20E−09	1
		35,689	0.28	3.28	0.284	1.24E−26	3
		9883	0.39	3.05	0.385	1.20E−11	2
		8174	0.14	3.03	0.437	1.02E−08	2
		41,553	0.43	2.96	0.188	5.05E−51	1
		30,156	0.04	2.87	0.790	9.16E−02	1
		34,751	0.05	2.52	0.461	5.84E−05	3
		34,530	0.18	2.38	0.277	6.05E−14	2
4	Crossbred	30,819	0.07	14.39	0.372	1.81E−102	2
		14,465	0.02	5.10	0.536	9.90E−18	3
		3834	0.04	4.01	0.344	1.58E−27	2
		36,398	0.18	4.00	0.288	1.25E−39	2
		31,018	0.48	3.52	0.281	7.71E−32	1
		6187	0.40	3.06	0.241	1.00E−32	3
		3420	0.43	2.99	0.271	2.72E−24	3
		43,184	0.08	2.77	0.282	7.68E−19	4
		33,882	0.07	2.09	0.290	1.63E−09	3
		4012	0.13	2.00	0.258	4.15E−11	1

*MAF* minor allele frequency, *SE* standard error

^a^Anonymous SNP identifiers were used to protect the intellectual property of The Maschhoff’s, LLC

Additional file 3: Table S3 contains all positional candidate genes and quantitative trait loci that are identified in pigs (AnimalQTLdb [39]) and humans (NHGRI-EBI GWAS catalog [41]) and located within 100 kb upstream or downstream of the GPSM significant SNPs in the Duroc, Landrace, Yorkshire, and crossbred populations. Eight positional candidate genes were identified in all four populations. Specifically, the *STX11* and *UTRN* genes were identified on chromosome 1, *AP3B2*, *FSD2*, *HOMER2*, and *WHAMM* on chromosome 7, and *TMEM132D* and *U6* on chromosome 14. Moreover, 14 positional candidate genes were identified in the Duroc, Landrace, and Yorkshire populations. More specifically, the genes *PRKN* on chromosome 1, *GALNT17* on chromosome 3, *CRSP2* and *ZDHHC17* on chromosome 5, *U6* on chromosome 6, *DDIT4L* and *EMCN* on chromosome 8, *ASNS*, *DGKB*, *GLCCI1*, *MIOS*, *RELT*, *UMAD1* on chromosome 9, and *PLD5* on chromosome 10 were located within 100 kb upstream or downstream of significant GPSM SNPs in each of the three purebred populations.

## Discussion

Polygenic selection on quantitative traits, induces small changes in allele frequencies at numerous loci across the genome over time [3, 14, 43]. The focus of this study was on the detection of the polygenic selection due to artificial selection over time for traits with complex architectures. The increasing abundance of genomic information from SNP arrays [44] has allowed many researchers to study changes in genotypic and allelic frequencies in commercial and indigenous global pig populations [5, 6, 16, 45, 46]. The rapid increase in the number of studies in this area has given rise to new analytical methods to detect large and small signatures of selection in the genome of commercially-reared livestock species, such as GPSM [14, 15]. In the present study, GPSM was used to estimate variance components and SNP genotype associations with the dependent variable AGE, which was calculated as the difference in months from January 2006 in a large commercial population of pigs that comprised three distinct pure populations (Duroc, Landrace, and Yorkshire) and a crossbred population that comprised the three pure populations. We found that the genomic relationship matrix accounted for confounding due to pedigree and population structure consistently across seven gene drop simulations, with false positive rates ranging from 0 to 0.015% (Table 10; see Additional file 2: Table S2).

The proportion of variation in age explained by the GRM ranged from 0.81 to 0.94. Based on simulations of genotypes from random mating versus selection analyzed with GPSM, Rowan et al. [14] stated that PVE is a function of the number of generations of selection, the number of total crosses per generation, and the genotype sampling scheme (even or uneven across generations). Our results are consistent with these conclusions as similar results across univariate variance component analyses using real and simulated genotype data indicated that pedigree structure and the distribution of AGE were the main determinants of PVE (Tables 5, 6). The difference between the gene-drop simulation PVE and the observed PVE was small for the Landrace and Yorkshire populations but was equal to 0.11 for the Duroc population. The main difference between simulated and observed data was the presence of selection, suggesting that the Duroc population was under stronger selection compared to the Landrace and Yorkshire populations. Selection indices for Duroc terminal populations generally consist of traits related only to growth, carcass and feed consumption, while selection indices for maternal lines consist of the previously stated traits and additional traits related to maternal prolificacy. Selecting on more traits means slower change for individual traits and their causal variants, especially when these traits are lowly heritable and require large amounts of data for accurate genetic evaluations. Thus, the overall genetic merit likely improved at a slower pace in the maternal populations compared to the Duroc population as a result of the added traits in the selection index. Furthermore, Rowan et al. [14] found smaller PVE across three cattle populations [PVE = 0.52, 0.59, and 0.46 in Red Angus (n = 15,295), Simmental (n = 15,350), and Gelbvieh (n = 13,031) populations, respectively] of similar sample sizes to the purebred populations in the current study. Differences between cattle and pigs in overall structure of the genetic selection programs related to the above factors likely contributed to the large difference between the PVE reported by Rowan et al. [14] and those found here.

The estimation of genetic correlations between pairwise combinations of the Duroc, Landrace, Yorkshire, and crossbred populations confirmed our assumptions on the similarity (or dissimilarity) between populations in their demographic and selection histories. A genetic correlation of nearly 1 for AGE between two populations suggests a high proportion of autosomal loci that are statistically associated with AGE undergoing similar changes in allelic frequency over time, while a genetic correlation between 0 and -1 suggesting the contrary (dissimilar or antagonistic changes in allele frequency in SNPs associated with AGE over time). The results of the simulation analysis, where randomly generated founder pig SNP genotypes are randomly dropped through the real pedigree of each population (mimicking genetic drift), validate this assumption, since the genetic correlations between populations were not significantly different from 0 (regardless of the most recent common ancestor in simulations) based on the likelihood ratio test (*P* > 0.05; Table 8). Selection objectives within The Maschhoffs are highly similar between the Landrace and Yorkshire populations and are the most dissimilar between the Duroc and each of the Landrace and Yorkshire populations. Estimated genetic correlations in the present study followed this pattern, as the estimated genetic correlations between the two maternal breeds were higher than those estimated between the Duroc population and either the Landrace or Yorkshire populations (Table 7). However, across all four populations, the genetic correlations were significantly higher than 0, indicating that the loci under selection are similar across populations. This is supported by the GPSM associations, as most strong associations were identified in multiple populations (Fig. 7) and there was a general increase in the number of associations when pooling populations (subsets 5 through 15; Table 9).

In our study, GPSM identified hundreds of SNPs that are significantly associated with AGE (*Q* < 0.10) in most populations (Table 9). There was a wide range in the number of pigs in each subset used in the GPSM analyses (Table 9). The GPSM method, as stated above and in other studies [14, 15], is a genome-wide association analysis, which is more powerful for the detection of SNP genotypes associated with a particular phenotype as the number of samples in the population increases, due to the increased precision in estimating SNP effects at a particular marker [44]. This inherent attribute of genome-wide association studies contributed to the large differences in the number of significant associations between SNP genotypes and AGE across subsets, as the number of significant SNPs showed a general increase with sample size (Table 9). However, this is only the case if the same loci are increasing in frequency across the different populations. The overwhelming majority of autosomal SNPs for each subgroup were not associated with AGE, according to the GPSM results (98.2 to 99.9% of the autosomal loci; Table 9). However, GPSM detected several SNPs that were significantly associated with AGE on each chromosome (Fig. 5). In addition, the nature of the genome-wide associations with AGE indicates that selection in these populations is likely polygenic (Fig. 5e–f).

The distribution of the age of the genotyped samples affects the power (false negative rate) of the GPSM analyses, with more even sampling providing more power and uneven sampling decreasing power [14]. The ages of the genotyped samples in this swine data are more evenly distributed across time than for many of the analyzed cattle datasets [14]. This may explain why we identified a relatively large number of selected loci with a moderate density SNP array. Our gene drop simulations, in agreement with the simulations of Rowan et al. [14], show that uneven sampling across time has a negligible effect on false positive rates.

A number of SNPs were detected by GPSM across at least two populations (Fig. 7). Visual assessment of the Manhattan plots of GPSM *Q*-values for each population allowed us to identify several regions along the autosomal genome that expressed similar patterns of GPSM significance across populations (Fig. 5). Of particular interest, are the four candidate genes (*MIOS*, *RPA3*, *UMAD1*, and *GLCCI1*) identified in the region of chromosome 9 that is associated with selection in all three purebred populations, which are all differentially expressed in ovarian tissues [47]. Most notably, the *MIOS* gene, which is commonly referred to as the “missing oocyte gene”*,* is well known for its role in regulating meiosis during oocyte development. In a study using *Drosophila*, a mutation in the *MIOS* gene caused erroneous oocyte development, i.e. instead of stimulating progression through each stage of meiosis, the described mutation caused oocyte progression towards polyploid nurse cells as opposed to fully functional, mature haploid gametes [48]. While there are no known studies that have evaluated the impact of mutations in the “missing oocyte” gene in pigs, our results suggest that selection pressure in The Maschhoff’s genetic program has had a significant effect on regions of the pig genome that influence fertility. As a litter-bearing species, pig breeders routinely place selection pressure on litter traits such as total number born and number born alive, especially in Landrace and Yorkshire pig populations. In addition, not only does selection of young replacement animals influence allele frequencies at quantitative trait loci, but the decisions on which animals to cull likely have similar effects. For example, gilts or sows in breeding populations that fail to express estrus cyclicity, conceive or farrow litters, or return to estrus within a reasonable period post-weaning are typically removed from the herd. It is likely that selection or culling of breeding animals due to reproductive performance and fertility issues, respectively, caused changes in allele frequency at loci near these four genes on chromosome 9. In addition to the *MIOS* gene, two genes of particular interest, *HOMER2* and *WHAMM*, were identified near significant GPSM SNPs on chromosome 7 in all four populations. In humans, both these genes are associated with lung function. However, the *HOMER2* gene is also associated with traits related to human body mass index. In addition, these two genes are located in regions of the pig genome that are associated with carcass traits such as backfat thickness, loin muscle depth and area, carcass length, dressing percentage, and estimated carcass lean content. The *HOMER2* and *WHAMM* genes were likely identified in each population due to the strong emphasis placed on carcass feed efficiency and lean meat production in selection indices for the Duroc, Landrace, and Yorkshire populations of the current swine breeding company. However, whether the *HOMER2* and *WHAMM* genes influence carcass traits through their effect on lung function (healthier pigs) or whether they have carcass-specific effects in swine is not known. Thus, further quantitative trait association studies and bioinformatics analyses are required to test these alternatives. The region containing the *UTRN* and *STX11* genes had AnimalQTL annotations related to white blood cell counts and virus titers (immunity) as well as adiposity measures (production). Genome-wide association studies in humans have shown that the *UTRN* and *STX11* genes are associated with lung function [49] and pre-treatment viral load in HIV-1 infection [50], respectively. Interestingly, the combination of the effects of production and immunity may also affect this locus on chromosome 1, suggesting that loci affecting production and immunity might be common targets of the selection across breeds.

The detection of significant associations across the autosomal genome in each of the Duroc, Landrace, Yorkshire, and crossbred populations indicates that artificial selection has influenced numerous genes in each of these populations of pigs. Furthermore, for the power to increase when pooling data across populations and shared signal across populations, common causal variants (or at a minimum, causal genes) must be segregating in the populations, and the variants must be responding to similar selection objectives. Thus, concordant traits across selection indices for maternal and terminal pig breeds are likely influenced by the same quantitative trait loci in the genome of each breed.

We confirmed that GPSM is robust in separating the changes in allele frequency due to genetic drift and artificial selection, through simulations. In each of the 11 gene-drop simulations, GPSM found very few spurious associations between SNP genotype and AGE (Table 10). Rowan et al. [14] identified false positives as significant at a rate of one SNP per 100,000 tests, which is similar to our results.

Except for two outliers [SNP 30819 in the crossbred population (14.39 months) and SNP 3420 in the Duroc population (13.32 months); Table 11], the absolute values of the SNP effects ranged from 0 to 8.21 months. The mean absolute values of SNP effects for AGE in significant associations were higher in the Duroc population (2.70 months) than in the other two purebred populations (1.66 and 1.55 months for the Landrace and Yorkshire populations, respectively). This suggests that the selection intensity is greater in the Duroc population, which induces larger changes in allele frequency over shorter periods of time than in the maternal breed populations. Selection in the Duroc population within The Maschhoff’s has focused on traits that increase the efficiency of terminal commercial progeny, such as increased growth and feed efficiency, decreased backfat depth, and increased carcass lean content. In general, the genetic predictions for growth and carcass traits are more accurate due to their moderate to large heritabilities, which increases selection response compared to those for maternal traits such as number of piglets born alive and litter weaning weight (traits that are emphasized in The Maschhoff’s Landrace and Yorkshire populations). Moreover, as stated previously, the maternal selection indices consisted of more traits, which could have decreased the rate of genetic progress for any single trait relative to an index consisting of fewer traits. This difference in breeding objective between the two groups of genetic lines is likely responsible for the larger SNP effects for AGE in the Duroc pigs. Crossbred pigs in The Maschhoff’s genetic selection program are not exposed to direct selection pressure. Instead, artificial selection occurs in the three genetic lines that constitute the genetic makeup of the crossbred population. Mean absolute values of the effects of SNPs for AGE that were in significant associations in the crossbred pigs (1.80 months) were similar to values reported for the three pure populations, suggesting that selection in the three purebred populations also changes allele frequencies in the crossbred population at similar rates. However, it must be noted that the genotype samples from the crossbred population were collected over a period of about four years compared to about 10 years for those from the three purebred populations (Table 2). We calculated mean yearly change in allele frequency for GPSM significant SNPs in each population, and the results for the three purebred populations were similar (0.018, 0.019, and 0.019 for the Duroc, Landrace, and Yorkshire populations, respectively). The mean yearly change in allele frequency for significant SNPs in the crossbred population was considerably larger than that in the purebred populations (0.024 vs. 0.018 to 0.019 per year). The ranking of the values of mean yearly allele frequency change for significant SNPs in each population differed from that of mean absolute values for SNP effects. This difference in results is likely due to the adjustment to SNP effects by inclusion of the genomic relationship matrices in the GPSM models, which allows for a more robust estimation of single-SNP selection proxies and more well-calibrated P-values than single-SNP regressions of year on allele frequency.

## Conclusions

We evaluated generation proxy selection mapping as an analytical method for detecting large and small signatures of artificial selection in a large commercial population of pigs from three purebred populations and one crossbred population. Numerous significant SNPs were detected across the genome in each genetic line, indicating that GPSM is effective to detect changes in pig genomes due to polygenic selection over relatively short time scales (~ 4 to 10 years). In addition, simulations proved that GPSM is well-calibrated to distinguish between changes in allele frequency over time resulting from genetic drift or artificial selection. Several SNPs were identified as significantly associated with AGE across multiple populations, which indicates that the selection objectives, genetic architectures, and causal variants underlying the quantitative traits that influence allele frequencies at loci are similar in each population over time. The results from this analysis and future analyses using GPSM will provide valuable insight into the biological mechanisms underlying selection on quantitative phenotypes in the commercial swine industry. Lastly, SNPs identified as being significantly associated with AGE have the potential to serve as indicators of genomic regions to prioritize in the development of genetic prediction models and selection schemes in swine breeding programs.

### Supplementary Information

**Additional file 1**: **Table S1:** Proportion of variation in AGE explained by SNPs for each purebred subset using five replications of randomly simulated genotype data.

**Additional file 2**: **Table S2:** Number of SNPs significantly associated with AGE for each subset using five replicates of randomly simulated genotype data.

**Additional file 3**: **Table S3:** Population, chromosome, SNP effect, *Q*-value, gene identifier, associated human traits, and associated pig traits for SNPs associated with AGE from GPSM analyses.

## Data Availability

Datasets supporting the conclusions of this article are available for non-commercial use via a data use agreement (DUA) with The Maschhoff’s, LLC.
